# A Novel Homozygous Missense Mutation of *PIEZO1* Leading to Lymphatic Malformation-6 Identified in a Family With Three Adverse Pregnancy Outcomes due to Nonimmune Fetal Hydrops

**DOI:** 10.3389/fgene.2022.856046

**Published:** 2022-05-13

**Authors:** Shuai Han, Xin Guo, Xiaogang Wang, Huijun Lin, Yiqi Yu, Jing Shu, Minyue Dong, Liwei Yang

**Affiliations:** ^1^ Center for Reproductive Medicine, Department of Obstetrics, Zhejiang Provincial People’s Hospital (Affiliated People’s Hospital, Hangzhou Medical College), Hangzhou, China; ^2^ Cancer Center, Department of Hematology, Zhejiang Provincial People’s Hospital (Affiliated People’s Hospital, Hangzhou Medical College), Hangzhou, China; ^3^ Center for Laboratory Medicine, Department of Clinical Laboratory, Zhejiang Provincial People’s Hospital (Affiliated People’s Hospital, Hangzhou Medical College), Hangzhou, China; ^4^ Center for Reproductive Medicine, Department of Reproductive Endocrinology, Zhejiang Provincial People’s Hospital (Affiliated People’s Hospital, Hangzhou Medical College), Hangzhou, China; ^5^ Department of Reproductive Genetics, Women’s Hospital, School of Medicine, Zhejiang University, Hangzhou, China

**Keywords:** lymphatic malformation-6, PIEZO1, whole exome sequencing, nonimmune fetal hydrops, a novel mutation

## Abstract

Lymphatic malformation-6 (LMPHM6) is a rarer form of nonimmune hydrops that often manifests as widespread lymphedema involving all segments of the body, namely, subcutaneous edema, intestinal/pulmonary lymphangiectasia, chylothoraces, and pleural/pericardial effusions. Here, we detected one rare and previously unobserved homozygous missense variant in *PIEZO1* (c.5162C>G, p.Ser1721Trp) as a novel genetic cause of autosomal recessive LMPHM6, in a family with three adverse pregnancy outcomes due to nonimmune fetal hydrops. Although, the loss-of-function mutations such as those usually including nonsense, frameshift, splice site, and also fewer missense variants in *PIEZO1* have been proved to lead to LMPHM6, among these, the biallelic homozygous mutations resulting in the loss of function of PIEZO1 have not been reported before. Here, we first strongly implicated impaired PIEZO1 function–associated LMPHM6 with a homozygous missense mutation in *PIEZO1*.

## Introduction

Lymphatic malformation-6 (LMPHM6; OMIM#616843) is a rare form of generalized lymphatic dysplasia (GLD) which often manifests as widespread lymphedema targeting all segments of the body, such as subcutaneous edema, intestinal/pulmonary lymphangiectasia, chylothoraces, and pleural/pericardial effusions (Connell et al., 2013). Notably, LMPHM6 often initially presents as nonimmune fetal hydrops, strikingly as facial and neck edema and pleural effusions (Fotiou et al., 2015).

PIEZO1 is a mechanosensitive ion channel that is mainly expressed in embryonic endothelial cells, such as the lymphatic vascular and red blood cells, following exposure to fluid pressure and flow (Choi et al., 2019; Gudipaty et al., 2017; Ranade et al., 2014). Previous studies have associated loss-of-function mutations in *PIEZO1* with autosomal recessive LMPHM6 (Fotiou et al., 2015; Lukacs et al., 2015). LMPHM6-associated *PIEZO1* mutations have been implicated in decreased PIEZO1 function with no calcium influx, resulting in reduced mechanosensitivity; a critical regulator of lymphatic vascular development, maturation, and formation (Cha et al., 2016). Conversely, the gain-of-function mutations in *PIEZO1* has been associated with autosomal dominant dehydrated hereditary stomatocytosis (DHS, OMIM#194380) (Albuisson et al., 2013). Moreover, previous studies have shown that DHS-associated mutations induce excessive calcium influx into the red blood cells, a phenomenon that is subsequently followed by cell dehydration (Cahalan et al., 2015). Intriguingly, DHS is mainly characterized by varying numbers of stomatocytes on peripheral blood smears which also occasionally presents with perinatal edema, which overlaps with the phenotype of LMPHM6 (Nonomura et al., 2018). This phenotypic heterogeneity, coupled with overlapping, complicates etiological diagnosis of nonimmune fetal hydrops. Moreover, studies have confirmed that most common genetic causes of nonimmune fetal hydrops are alpha-thalassemia related, especially in Southeast Asia (Lorey et al., 2001), and only a handful of genetic causes have been found to be predisposing factors to nonimmune fetal hydrops. These include metabolic disorders such as infantile sialic acid storage disease (OMIM#269920) (Lefebvre et al., 1999), mucopolysaccharidosis type VII (OMIM#253220) (Nelson et al., 1982), glycogen storage disease IV (OMIM#232500) (Cox et al., 1999), and LMPHM6 (Fotiou et al., 2015). Identification of these accurate molecular genetic diagnostic markers are imperative in understanding the etiology of and to accurately distinguish DHS from other diseases that cause nonimmune fetal hydrops.

Herein, we performed whole exome sequencing (WES) and identified a novel homozygous missense mutation in a mechanically activated ion channel, PIEZO1, in a family with three adverse pregnancy outcomes due to nonimmune fetal hydrops. The couple’s adverse pregnancy history, coupled with impaired PIEZO1 expression, suggested that c.5162C>G in *PIEZO1* is a loss-of-function mutation that causes LMPHM6.

## Methods and Materials

### Chromosomal Karyotyping and Molecular Genetic Analysis

Amniotic fluid was obtained by amniocentesis for karyotyping and molecular genetic analysis. Amniotic fluid cells were cultured and analyzed by G-banding at a resolution of 400 bands on average following the standard procedures to confirm the approximate location of a gain or loss of more than 5 Mb. Genomic DNA was extracted from the amniotic fluid cells using the QIAGEN QIAamp DNA Blood Mini kit (Qiagen, Hilden, Germany). For chromosomal microarray analysis (CMA), the genomic DNA was digested, ligated with adaptors, amplified, purified, labeled with biotin, and hybridized to the Affymetrix CytoScan 750K Array (Affymetrix, Santa Clara, CA, United States), then it was washed with Affymetrix GeneChip Fluidics Station 450 and scanned with an Affymetrix GeneChip Scanner 3000 to detect copy number variants (CNVs), loss of heterozygosity (LOH), uniparental disomy (UPD), and identity by descent (IBD). For WES, DNA libraries were constructed, which were subjected to whole-exome capture on an Agilent SureSelect Human All Exon V6 Capture (Agilent, California, United States). Subsequently, we performed high-throughput sequencing on an Illumina HiSeq 2000 (Illumina, Inc., San Diego, CA, United States) to analyze point mutations and small deletion/insertions. All the detected sequence variants were systematically evaluated and classified according to the standards and guidelines recommended by the American College of Medical Genetics and Genomics (ACMG) (Richards et al., 2015).

### Polymerase Chain Reaction–Based Sanger Sequencing

To further validate the WES results, primers (F-TCATCCTCAACCACATGGTCA/R-GACGAT GGCCGTCATCCAG) for *PIEZO1* c.5162C>G were designed according to the *PIEZO1* sequence (NM_001142864.4). Polymerase chain reaction (PCR) was performed according to the following procedure: 95°C 5 min, 32× (95°C 30 s, 59°C 30 s, 72°C 30 s), 72°C 15 min, the products of which were sequenced and subsequently analyzed with CHROMAS.

### Bioinformatics Analysis

Sorting Intolerant from Tolerant (SIFT, http://sift-dna.org), Polyphen-2 (http://genetics.bwh.harvard.edu/pph2/), and MutationAssessor were used to predict the effect of the missense site on PIEZO1.

### Blood Smear Microscopy

Blood smear microscopy of the umbilical cord blood was carried out with the benefit of May-Grunwald–Giemsa staining.

### Real-Time Polymerase Chain Reaction

We isolated total RNA from the muscle cells using TRIZOL reagents. We reversely transcribed 1 μg of the total RNA to cDNA by random hexamers using M-MLV reverse transcriptase (Fermentas, Vilnius, Lithuania). Real-time quantitative reverse transcription–PCR was done as described by Han et al. (2015). The sequences of the primers are as follows: *β*-actin-F: TGA​CGG​GGT​CAC​CCA​CAC​TGT​GCC​CAT​CTA; *β*-actin-R: CTA​GAA​GCA​TTT​GCG​GTG​GAC​GAT​GGA​GGG; PIEZO1-F: CTG​TAC​CAG​GGA​CTG​ATG​CG; PIEZO1-R: CGA​TGG​CCG​TCA​TCC​AGA​AG.

### Western Blotting

Total protein was extracted from the muscle tissue of the aborted fetus using the Minute™ Total Protein Extraction Kit (Invent, SD-001/SN-002). Western blotting was performed as described previously using the PIEZO1 monoclonal antibody (1:200, Proteintech, 15939-1-AP) and GAPDH monoclonal antibody (1:1,000, Beyotime AF1186) (Han et al., 2015).

## Results

A 25-year-old woman (gravidity 4, parity 0) in 20 + 3 weeks of gestation was referred to our hospital, with an ultrasonography showing fetal pleural effusion. Her family history revealed that she had her two previous adverse pregnancy outcomes due to nonimmune fetal hydrops. These had occurred at 36 and 28 weeks of pregnancy and had led to immediate termination of pregnancy in a local hospital. Then, for the first hydropic fetus, chromosome karyotype analysis of the umbilical cord blood revealed a 46,XY karyotype; BACs-on-Beads (BoBs) assay was performed on the aborted fetal tissues, with no meaningful microdeletions and microduplications. For the second hydropic fetus, the chromosome karyotype analysis of the amniotic fluid cells revealed a 46,XY karyotype; CMA was performed on the aborted fetal tissues, with no abnormal results. Moreover, appearance of the two aborted fetuses supported generalized fetal hydrops, especially face and neck, without cardiac involvement. During her first pregnancy, she had undergone induced abortion at 7 weeks of gestation due to cultural reasons.

**FIGURE 1:**
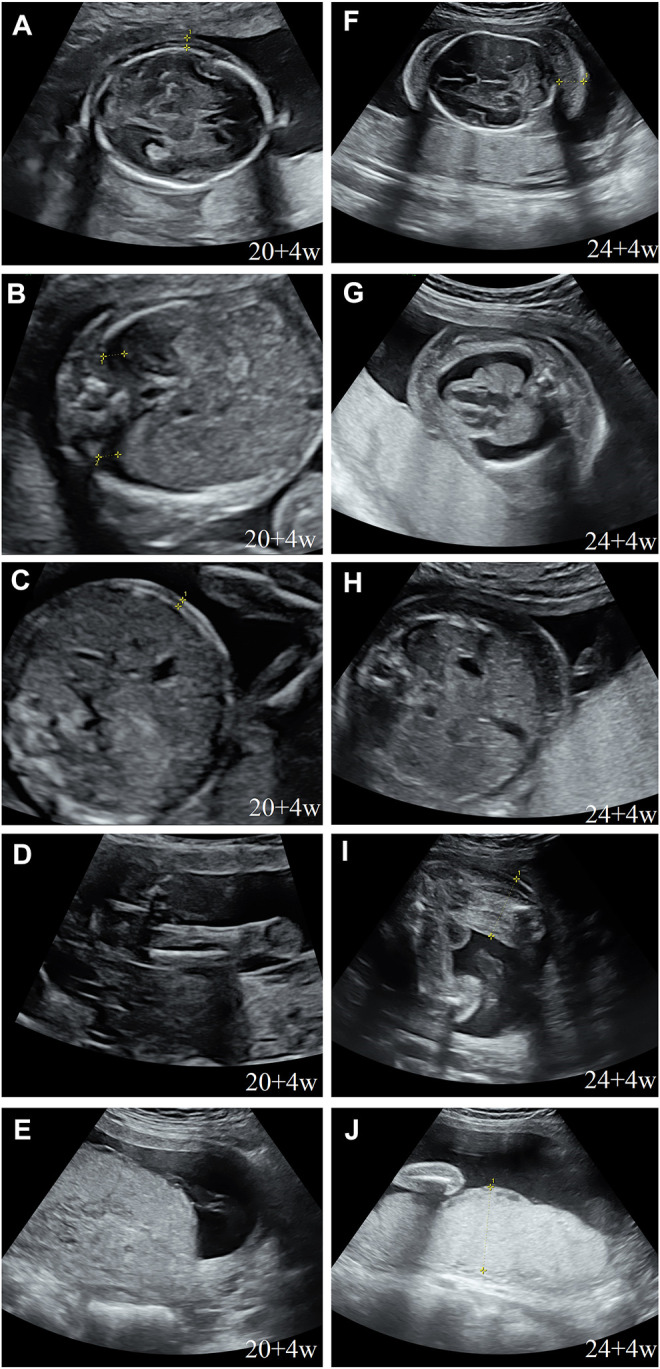
Prenatal ultrasound. **(A)** Thalamus transverse section: subcutaneous edema: 3 mm; **(B)** thorax transverse section: bilateral hydrothorax: 4 mm; **(C)** abdominal transverse section: no subcutaneous edema; **(D)** sagittal section of the thigh: no subcutaneous edema; **(E)** no placental thickening; **(F)** cerebellum transverse section: subcutaneous edema: 14 mm; **(G)** thorax transverse section: subcutaneous edema: 10 mm, bilateral hydrothorax: 10 mm, and lung collapse, heart pressed; **(H)** abdominal transverse section: subcutaneous edema: 10 mm; **(I)** sagittal section of the thigh: subcutaneous thickening: 9 mm; and **(J)** placental thickening: 53 mm.

**FIGURE 2:**
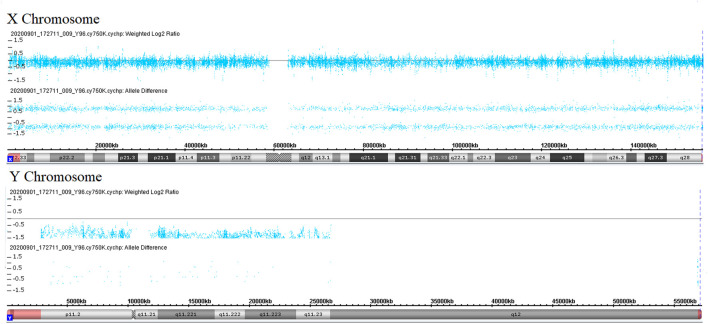
Copy number analyses *via* CMA. Weighted log2 ratio: values of tested SNP log signal intensity compared to the reference sample SNP intensity are processed through a Bayes wavelet shrinkage estimator, and then these processed values are input to the CNState algorithm HMM; intensity ratio of 0 = 2 copies; intensity ratio of −1 = 1 copies; values of chromosomes X and Y are centered below 0 and −1, respectively, indicating sex chromosome mosaicism. Allele difference is computed based on differencing A signal and B signal; genotype “AA”: (0.5 + 0.5)–(0) = 1; genotype “AB”: (0.5)–(0.5) = 0; genotype “BB”: (0)–(0.5 + 0.5) = −1; values of chromosome X indicates maternal uniparental disomy of chromosome X.

During her fourth pregnancy, first-trimester ultrasonography revealed 1.4-mm nuchal translucency (NT), and noninvasive prenatal testing (NIPT) indicated that the fetus was at low risk for trisomy 21/18/13. A more detailed ultrasonography, performed at our hospital at 20 + 4 weeks of gestation, revealed subcutaneous edema along the head:3 mm and bilateral pleural effusion: 4 mm (Figures 1A,B). And there was no subcutaneous edema along the trunk and limbs, and no placental thickening (Figures 1C-E). Maternal immunological serology, infections, and cardiac ultrasonic inspection revealed no abnormalities. And the blood count, iron balance, hemolytic indices (Table 1), and hemoglobin electrophoresis in the couple showed no indication of hereditary thalassemia. Meanwhile, the woman underwent amniocentesis, which subsequently revealed a G-band karyotype of 46,XY (23) and 46,XX (77). CMA also further revealed XY (25%) mosaicism with maternal uniparental disomy of chromosome X (UPD (X) mat) (Figure 2). However, previous studies have shown that neither UPD of the X chromosome nor sex chromosome mosaicism can lead to nonimmune fetal hydrops (del Gaudio et al., 2020).

**TABLE 1:** Laboratory values of the blood count, the hemolytic indices, the iron balance, and IgG antibody A(B) or antibody D.

Parameters		II 1	II 2
blood count	RBC (10^12^/L)	4.25	5.1
	Hb (g/L)	118	132
	Htc	0.356	0.363
	MCV (fL)	83.1	85.9
	MCH (pg)	28	30
	MCHC (g/L)	335	318
	WBC (10^9^/L)	5.3	6.9
	PLT (10^9^/L)	225	189
iron balance	Serum Fe (µmol/L)	29.1	
	Ferritin (µg/L)	17.4	
	TIBC	49	
	TSAT(%)	35	
	Hepcidin (µg/L)	98	
	Hepcidin/ferritin ratio	1.8	
	sTfR	2.3	
hemolytic indices	Reticulocytes	2%	
	Bilirubin	11	
	Haptoglobin	1.1	
	LDH	2.5	
	Urine Rous test	negative	
Rh-alloimmunisation	anti-D immunoglobulin	negative	
ABO-alloimmunisation	Anti-A(B) immunoglobulin	64	

Normal control ranges: red blood cell count, RBC (female: 3.8–4.9 × 10^12^/L; male: 4.3–5.7 × 10^12^/L); hemoglobin, Hb (female: 120–160 g/dL; male: 130–180 g/dL); hematocrit, Htc (0.350–0.450); mean corpuscular volume, MCV (78–100 fL); mean corpuscular hemoglobin, MCH (27–34 pg); mean corpuscular hemoglobin concentration, MCHC (316–354 g/L); white blood cell count, WBC (3.5–9.5 × 10^9^/L); platelet, PLT (100–300 × 10^9^/L); serum Fe (10.6–28.3 μmol/L); ferritin (4.6–204 μg/L); total iron binding capacity, TIBC (54–77 μmol/L), transferrin saturation, TSAT (21–48%); hepcidin (13.1–104.8 μg/L); hepcidin/ferritin ratio (0.2–2.2); soluble transferrin receptor, sTfR (1.9–4.4 mg/L); reticulocytes (0.5–3.0%); bilirubin (0–23 μmol/L); haptoglobin (0.3–2 g/L); lactate dehydrogenase, LDH (2.34–4.68 μkat/L); urine Rous test (negative); anti-D, immunoglobulin (negative); anti-A(B) immunoglobulin (8–64).

**FIGURE 3:**
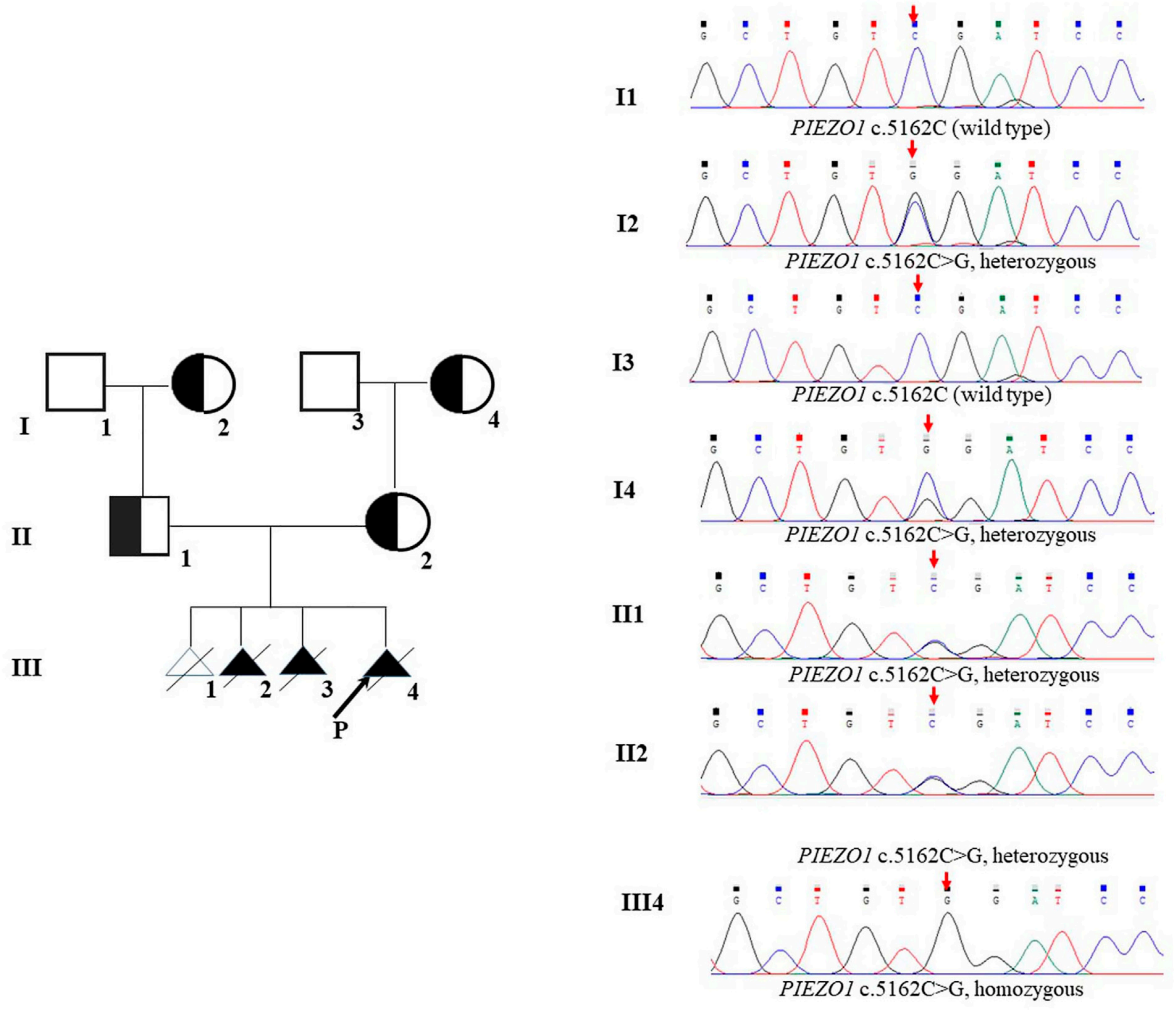
Pedigrees and DNA sequences. The arrow in III shows the proband. The red arrows indicate the mutations.

**FIGURE 4:**
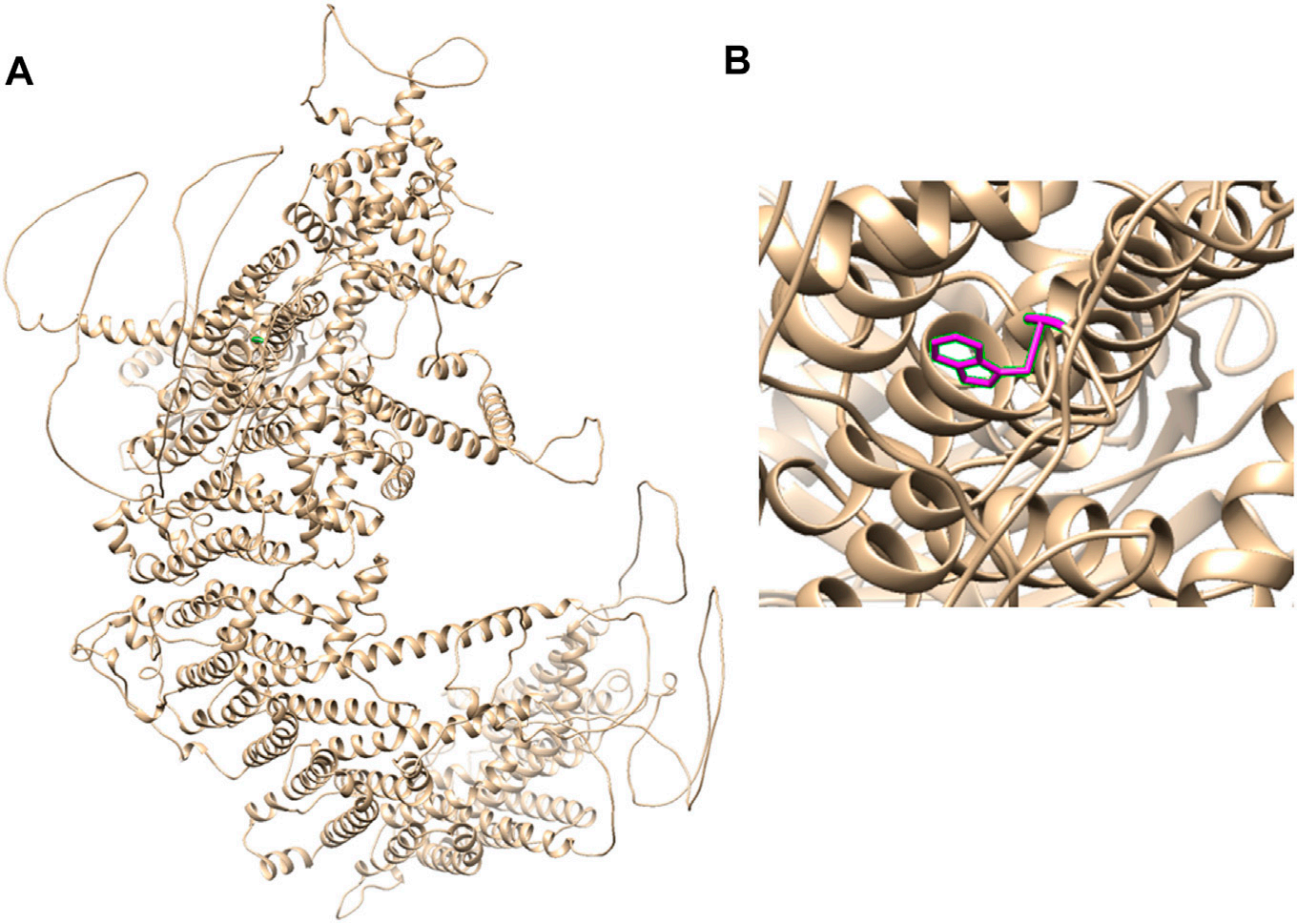
3D modeling of *PIEZO1* p.Ser1721Trp. **(A)** Ribbon modeling of PIEZO1 (1–2,521). **(B)** Close-up of *PIEZO1* p.Ser1721Trp is depicted in magenta.

**FIGURE 5:**
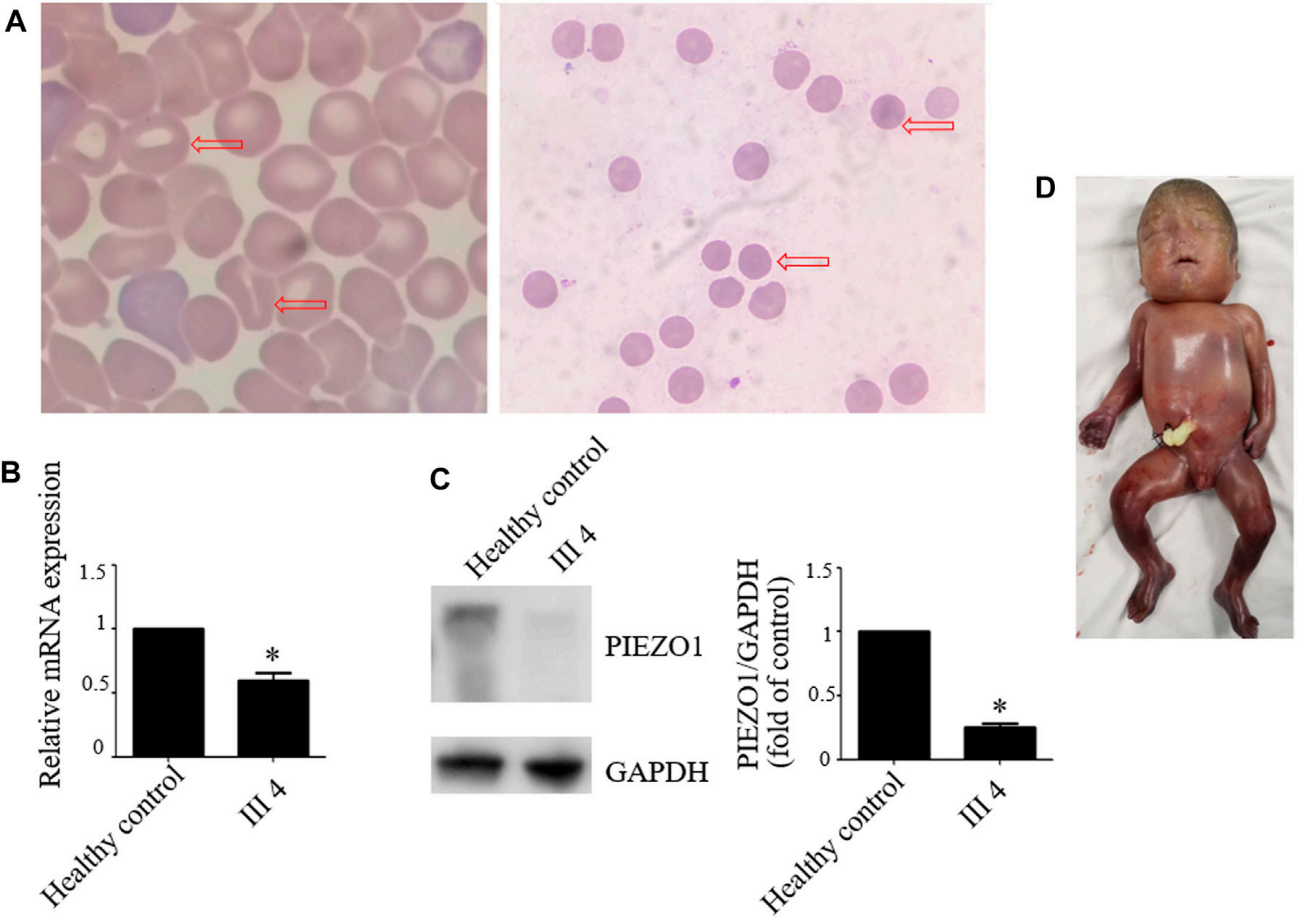
Blood smear microscopy, PIEZO1 protein expression and the aborted fetus. **(A)** Blood smear microscopy showing occasional stomatocytes and spherocytes (red arrows) in III 4. **(B)** PIEZO1 mRNA expression is defective in III 4. **(C)** PIEZO1 protein expression is defective in III 4. **(D)** The appearance of the aborted fetus was male and manifested generalized subcutaneous edema, especially face and neck swelling.

Therefore, we performed WES, requested due to the recurrent hydrops, and found a homozygous missense in *PIEZO1*: c.5162C>G (p.Ser1721Trp), with the validation of Sanger sequencing (Figure 3). Previous studies have associated biallelic PIEZO1 mutations with LMPHM6, with a close relationship to nonimmune fetal hydrops (Fotiou et al., 2015). Sanger sequencing results also confirmed that the couple was heterozygous for c.5162C>G (Figure 3). Analysis extension to the first and second sib was not performed due to loss of DNA sample. Alignment of the human PIEZO1 protein with homologs from human and other species indicated that p.Ser1721Trp was a nonconservative substitution. This was also predicted to be deleterious by SIFT, PolyPhen-2, and MutationAssessor. The variant was not recorded in HGMD (http://www.hgmd.cf.ac.uk/ac/index.php) or ClinVar (https://www.ncbi.nlm.nih.gov/clinvar/) and has never been described in any literature. Allele frequency is not available in the Genome Aggregation Database (gnomAD) (http://gnomad.broadinstitute.org/), Exome Aggregation Consortium (ExAC) (http://exac.broadinstitute.org/), and in the 100 healthy subjects who were analyzed. 3D modeling of *PIEZO1* p.Ser1721Trp is depicted in Figure 4. The results from blood smear microscopy of the umbilical cord blood revealed occasional stomatocytes and spherocytes, indicating subtle abnormalities (Figure 5A). We also performed blood smear microscopic analysis on the couple, due to the hematological features described in *PIEZO1* heterozygous asymptomatic carriers (Andolfo et al., 2013), and it was, however, normal in the couple. Moreover, there was no abnormal values associated with maternal red cell alloimmunization, essentially meaning Rh-alloimmunization and ABO-alloimmunization (Table 1), further excluding immune fetal hydrops (Devendra et al., 2021). To further evaluate the pathogenic role of *PIEZO1* variants, we analyzed PIEZO1 expression in the proband and alongside healthy controls. RT-PCR analysis demonstrated that PIEZO1 expression is downregulated in the proband. Western blotting analysis from muscle cells revealed a marked decrease of PIEZO1 protein in the proband when compared to the healthy controls with about 80% of expression, implicating PIEZO1 loss of function (Figures 5B,C). Finally, the p.Ser1721Trp variant is classified as a variant of uncertain significance according to the ACMG principles. PM2 was applied as it was absent in the gnomAD and ExAC databases. We applied PP3, as it was interpreted as “deleterious” by SIFT, PolyPhen-2, and MutationAssessor. We applied PP4 criterion, as the recurrence of nonimmune hydrops with negative standards and WES is highly indicative for a monogenic disorder, and the mutation segregated with the peculiar phenotype in the third affected fetus.

Next, we performed another senior ultrasonography and observed more severe and ongoing hydrops. These included pleural effusion (10 mm) and subcutaneous edema of the head (14 mm), truck (10 mm), limb (9 mm), and placental thickening (53 mm) (Figures 1G–J). After extensive genetic counseling, the couple chose to terminate the pregnancy at 24 + 4 weeks of gestation. The aborted fetus appeared male, and manifested generalized subcutaneous edema, especially on the face and neck (Figure 5D).

## Discussion

In this article, we have described a previously unobserved rare homozygous missense variant in *PIEZO1* (c.5162C>G, p.Ser1721Trp) as a novel genetic cause of autosomal recessive LMPHM6. Notably, a G-band karyotype revealed 46,XY (23) and 46,XX (77), while the CMA further revealed XY (25%) mosaicism with UPD (X) mat. So, what is the real genetic cause behind fetal edema? We speculate that nondisjunction in meiosis II leads to a failure of the X sister chromatids to separate into daughter cells, which can subsequently result in an isodisomic oocyte of the X chromosome. Following fertilization with a normal haploid Y gamete, the zygote is expected to exhibit trisomy rescue, either by losing the paternal chromosome Y giving rise to UPD X or by losing the maternal chromosome X giving rise to XY. However, neither has the maternal UPD for chromosome X been documented for imprinting genes nor has sex chromosome mosaicism been attributable to the specific phenotypes (del Gaudio et al., 2020). Subsequently, the phenotype of recurring nonimmune fetal hydrops and the peculiar ultrasound feature in the family guided the WES data analysis to a diagnosis of *PIEZO1* hydrops. Finally, the p.Ser1721Trp variant is classified as a “variant of uncertain significance” according to the ACMG principles. However, the analysis of the fetus’s muscle cells revealed greatly attenuated PIEZO1 expression, which strongly implicated impaired PIEZO1 function–associated hereditary lymphedema. Some further functional studies *in vitro* and *in vivo* about the ion channel function of PIEZO1 could be done to yield valuable insight into the pathogenesis of this condition, and then, the classification of the p.Ser1721Trp variant may be upgraded. Indeed, the couple were not close relatives, but they may be distant consanguineous, considering their parents and ancestors living in the same village. And also, seven missense mutations in *PIEZO1* (Leu939Met, Arg1925Trp, Gly2029Arg, Val2171Phe, Pro2430Leu, Arg2456Cys, and Phe2458Leu) have been demonstrated to lead to lymphatic malformation-6 (Fotiou et al., 2015; Lukacs et al., 2015; Mastromoro et al., 2021).

PIEZO1 acts as a mechanosensitive ion channel and encompasses 36 transmembrane domains. It is mainly expressed in non-sensory cell types such as the human erythrocytes and endothelial cells, where it not only plays a critical role in endothelial orientation, blood vessel formation, vascular structure, and regulation of urinary osmolarity but also acts as a sensor of cell tension (Gudipaty et al., 2017; Hyman et al., 2017; Li et al., 2014; McHugh et al., 2010). PIEZO1 was firstly associated with autosomal dominant DHS, characterized by mild to moderate hemolytic anemia with varying numbers of stomatocytes and spherocytes (Zarychanski et al., 2012). In the erythrocytes, the gain-of-function mutations in *PIEZO1* were implicated in dehydration of RBCs due to excessive calcium influx and potassium efflux (Cahalan et al., 2015). Lukacs et al. (2015) associated the phenotypic consequence of *PIEZO1* loss-of-function mutations with autosomal recessive LMPHM6; this was accompanied by persistent lymphedema caused by lymphatic dysplasia. LMPHM6, which is characterized by widespread lymphedema, appears as persistent bilateral pleural effusions, ascites, and subcutaneous edema, such as striking facial swelling (rarely seen in other forms of lymphedema). Mechanical stimulation such as oscillatory shear stress promotes lymphatic vascular development by triggering Wnt/*β*-catenin signaling in the lymphatic endothelial cells (Cha et al., 2016). Therefore, researchers have hypothesized that PIEZO1 regulates development of the lymphatic vasculature, as evidenced by a marked upregulation of PIEZO1 in the lymphatic vessels of human fetal peritoneum at gestational week 17 (Andolfo et al., 2013). LMPHM6-associated *PIEZO1* mutations causes PIEZO1, with no calcium influx, leading to hyperhydration of the red blood cells, which could be distinguished from DHS (Cahalan et al., 2015). Notably, some individuals with DHS also transiently present with perinatal edema, including either pericardial or pleural effusions and/or subcutaneous edema, albeit with no relationship to anemia (Entezami et al., 1996; Andolfo et al., 2013). LMPHM6-affected individuals and unaffected carriers may present mild hemolysis, with spherocytes and stomatocytes on blood smear (Andolfo et al., 2019). To date, however, the mechanism underlying this clinical overlap remains unclear. Fotiou et al. (2015) described several LMPHM6 patients with *PIEZO1* mutations in trans who showed occasional stomatocytes or mild, asymptomatic hemolytic anemia. In our study, the proband with homozygous missense variants presented with occasional stomatocytes and spherocytes, with subtler changes than those in DHS. Conversely, unaffected heterozygous individuals manifested no anemia, no spherocytes and stomatocytes on blood smear, and no decreased osmotic fragility. The presence of generalized fetal hydrops, especially on the face and neck, of the three aborted fetuses, coupled with the loss-of-function *PIEZO1* phenotype suggested for the first time a correlation between a homozygous missense mutation of PIEZO1 and LMPHM6.

In accordance to literature data, it is still ambiguous whether different forms of PIEZO1 exist for certain key pathogenic mechanisms, thus resulting in the DHS or LMPHM6 phenotypes, respectively. The distribution of *PIEZO1*-associated DHS or LMPHM6 mutations on PIEZO1 secondary structure are not regular. *PIEZO1* p.Ser1721Trp locates in one of the extracellular domains (1721–1729), nearing one of the transmembrane sequences 1700–1720, predicted by the TMHMM 2.0 web server (http://www.cbs.dtu.dk/services/TMHMM/).

Accumulation of excessive pathological fluid in more than two body cavities, namely, subcutaneous tissues, suggested that fetal hydrops should be considered. However, prenatal signs of nonimmune fetal hydrops may not be so specific, thus it must be distinguished from immune fetal hydrops caused by red cell alloimmunization. Generally, this condition not only affects 2.5 in every 10,000 pregnancies but can also be life threatening (Steurer et al., 2017). Etiologically, nonimmune fetal hydrops are characterized by monogenic diseases, chromosomal structural abnormalities, chromosomal copy number variations, infections, hematologic diseases, cardiovascular disorders, and extrathoracic tumors, as well as other causes, of which genetic causes have been documented to account for one-third (Norton et al., 2015). According to recently published literature, genetic testing with karyotype and/or CMA is effective in detecting a vast majority of genetic factors–associated fetal hydrops, involving chromosomal structural abnormalities and chromosomal copy number variations, which cannot detect variants in exons and introns of the genome (Sparks et al., 2019). Thus, a clear etiology of nonimmune fetal hydrops should be determined by WES, especially for our case. Zhou et al. (2021) demonstrated that WES should be offered to all hydropic fetuses, especially recurrent nonimmune fetal hydrops cases, regardless of whether hydrops are isolated or are in presence of structural abnormalities. Many monogenic disorders predisposing to nonimmune fetal hydrops (OMIM #236750) have been illustrated such as erythropoietic porphyria (*UROS*), Gaucher disease (*GBA*), infantile sialic acid storage disease (*SLC17A5*), mucopolysaccharidosis type VII (*GUSB*), glycogen storage disease IV (*GBE1*), and LMPHM6.

In summary, we identified a novel homozygous missense mutation: *PIEZO1* c.5162 C>G (p.Ser1721Trp) that leads to LMPHM6, in a family with three adverse pregnancy outcomes due to nonimmune fetal hydrops. Although, etiological heterogeneity in the nonimmune fetal hydrops may complicate diagnosis of the disease, combination of molecular genetic testing can somehow solve the mystery. The results of this study further affirms the association between the phenotype and genotype of LMPHM6 and provides the first theory of homozygous missense mutation in *PIEZO1*, resulting in the loss of function of PIEZO1.

## Data Availability

The datasets for this article are not publicly available due to concerns regarding participant/patient anonymity. Requests to access the data sets should be directed to the corresponding author.
